# The Peripheral Amyloid-β Nexus: Connecting Alzheimer’s Disease with Atherosclerosis through Shared Pathophysiological Mechanisms

**DOI:** 10.1007/s12017-025-08836-2

**Published:** 2025-03-03

**Authors:** Manal M. Khowdiary, Hayder M. Al-kuraishy, Ali I. Al-Gareeb, Ali K. Albuhadily, Ahmed A. Elhenawy, Eman K. Rashwan, Athanasios Alexiou, Marios Papadakis, Mohammed E. Abo‑El Fetoh, Gaber El-Saber Batiha

**Affiliations:** 1 Department of Chemistry, Faculty of Applied Science, Lieth Collage, Umm Al-Qura University, 24382 Makkah, Saudi Arabia; 2 Department of Clinical Pharmacology and Medicine, College of Medicine, Mustansiriyah University, Baghdad, Iraq; 3 Jabir Ibn Hayyan Medical University, Al-Ameer Qu./Najaf-Iraq, PO. Box13, Kufa, Iraq; 4 Chemistry Department, Faculty of Science, Al-Azhar University, Nasr City, Cairo 11884 Egypt; 5 Chemistry Department, Faculty of Science, AlBaha University, 65731 Al Bahah, Saudi Arabia; 6 Department of Physiology, College of Medicine, Jouf University, Akaka, Saudi Arabia; 7 Department of Research & Development, Funogen, 11741 Athens, Attiki Greece; 8 University Centre for Research & Development, Chandigarh University, Chandigarh-Ludhiana Highway, Mohali, Punjab India; 9 University Hospital, University of Witten-Herdecke, Heusnerstrasse 40, 42283 Wuppertal, Germany; 10 Department of Pharmacology and Toxicology, Faculty of Pharmacy, Egyptian Russian University, Badr City, 11829 Cairo Egypt; 11 Department of Pharmacology and Therapeutics, Faculty of Veterinary Medicine, Damanhour University, Damanhour, 22511 AlBeheira Egypt

**Keywords:** Alzheimer's disease, Atherosclerosis, Amyloid-beta (Aβ), Vascular dysfunction, Neuroinflammation, Oxidative stress, Insulin resistance, Aβ clearance pathways

## Abstract

Alzheimer’s disease (AD) and atherosclerosis (AS) are two chronic diseases with seemingly distinct pathologies. However, emerging research points to a bidirectional relationship driven by common mechanisms, such as inflammation, oxidative stress, and dysregulation of Amyloid-Beta (Aβ). This review focuses on the role of Aβ as a critical molecular link between AD and AS, emphasizing its contribution to neuronal impairment and vascular damage. Specifically, peripheral Aβ produced in the pancreas and skeletal muscle tissues exacerbates AS by promoting endothelial dysfunction and insulin resistance (IR). Furthermore, AS accelerates AD progression by impairing cerebral blood flow and inducing chronic hypoxia, causing Aβ accumulation. This review critically evaluates recent findings, highlighting inconsistencies in clinical studies and suggesting future research directions. Understanding the bidirectional influence of AD and AS could pave the way for novel therapeutic approaches targeting shared molecular pathways, particularly emphasizing Aβ clearance and inflammation.

## Introduction

**Fig. 1:**
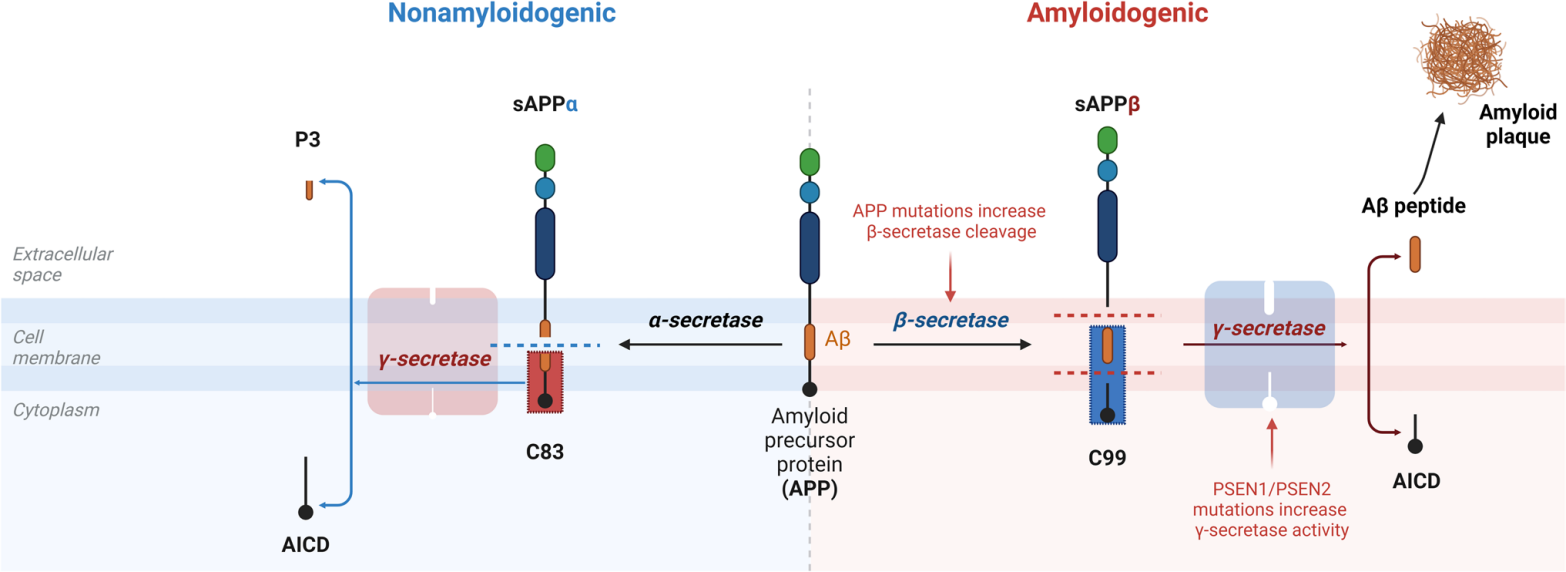
Processing of amyloid precursor protein: The γ and β secretases generate neurotoxic Aβ through the amyloidogenic pathway. β secretases convert amyloid precursor protein (APP) to the soluble amyloid precursor protein β (sAPPβ), which further proceeds to Aβ and APP IntraCellular Domain (AICD). Aβ further aggregated to form amyloid plaque. The non-amyloidogenic pathway generates sAPPα by α-secretase, forming P3 and AICD

Alzheimer's disease (AD) is a progressive neurodegenerative disease linked with cognitive impairment, memory dysfunction, and neuropsychiatric disorders (Scheltens et al., 2021). AD accounts for about 75% of all dementia types in old-age subjects (R. Li et al., 2022). There are two types of AD: sporadic AD accounts for 90% of AD and familial AD accounts for 10% of AD cases. Sporadic AD is more correlated with old age > 65 years and late-onset AD; however, familial AD is related to the development of early-onset AD (Jellinger, 2020). AD neuropathology is characterized by the progressive deposition of extracellular Aβ and intracellular hyperphosphorylated tau protein (Trejo-Lopez et al., 2022). Accumulated Aβ in the brain is attributed to either the overproduction of Aβ from mutant amyloid precursor protein (APP) or a defect in the clearance of Aβ (Y. Li et al., 2020). Under typical physiological settings, most of the APP processing, particularly in youth, occurs via the non-amyloidogenic route, resulting in the production of the neuroprotective soluble APP alpha (sAPPα). The amyloidogenic pathway is mainly facilitated by γ and β secretase enzymes, while the non-amyloidogenic pathway is facilitated by α secretase. In aging and chronic inflammatory and oxidative stress diseases, APP processing is redirected toward the amyloidogenic pathway, producing the neurotoxic Aβ_1-42_, which induces inflammation and neuronal cell death (Fig. 1) (Dar & Glazner, 2020; Pfundstein et al., 2022).

**Fig. 2:**
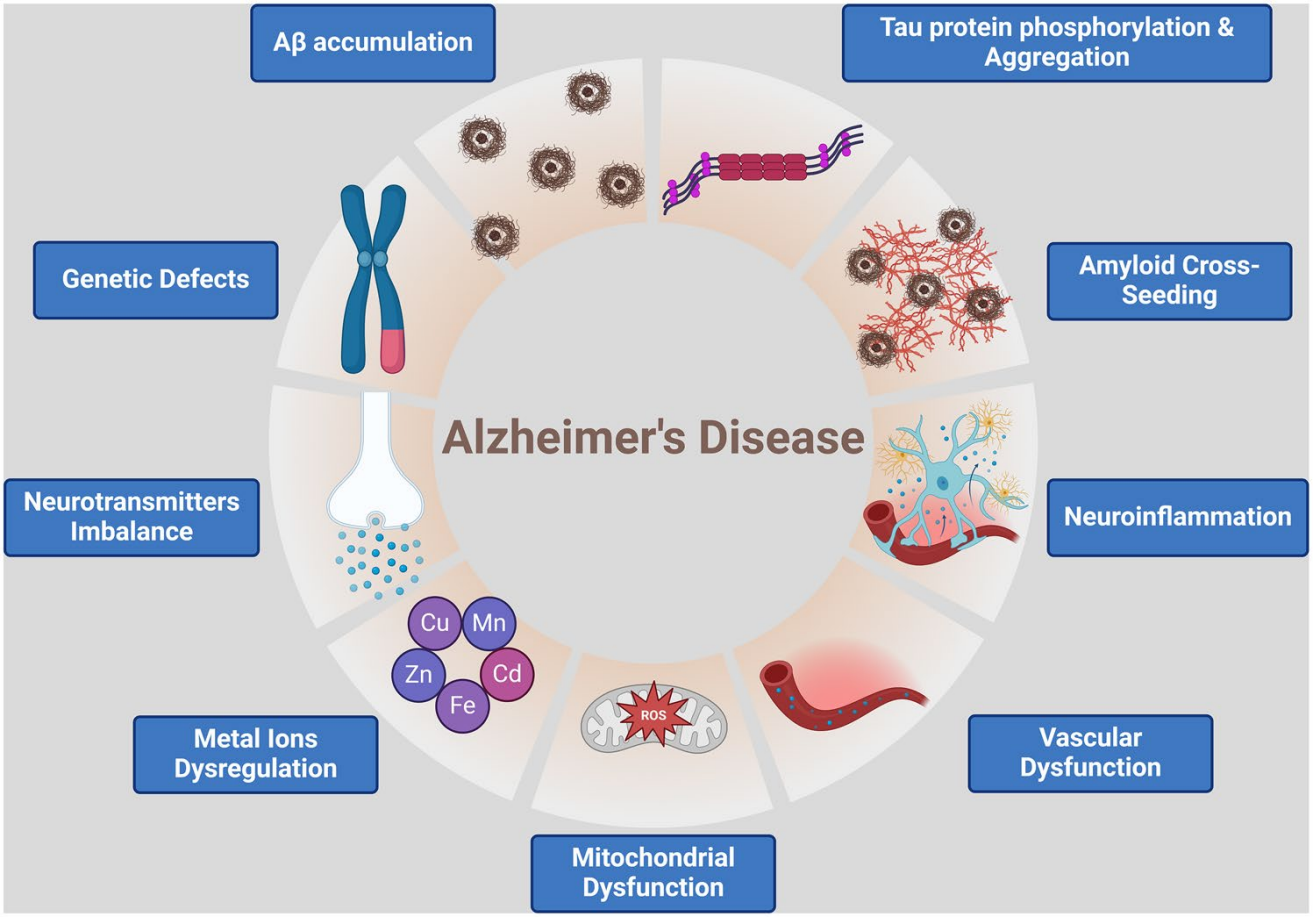
AD neuropathology: Genetic and environmental factors are implicated in the pathogenesis of AD by inducing progressive accumulation of Aβ and tau protein, which causes mitochondrial dysfunction and imbalance of neurotransmitter release. Seeding of Aβ in the brain triggers neuroinflammation and vascular dysfunction, which are involved in the pathogenesis of AD

Furthermore, tau protein is usually present in the healthy brain and is intricate in regulating axonal transport and microtubule stabilization (Muralidar et al., 2020). Nonetheless, genetic mutation of the tau protein gene or activation of the inflammatory signaling pathway induces hyperphosphorylation of the tau protein (Basheer et al., 2023). The accumulation of hyperphosphorylated tau protein, which forms neurofibrillary tangles (NFT), is associated with increasing neuronal damage and the onset of AD neuropathology (Basheer et al., 2023; Muralidar et al., 2020). Aβ and NFT interact together to induce inflammation and oxidative stress, which provoke neurodegeneration in AD (Zhang et al., 2021). Therefore, AD neuropathology is diverse and related to dissimilar cellular and sub-cellular disorders, such as autophagy dysfunction, mitochondrial dysfunction, oxidative stress, and neuroinflammation (Fig. 2) (Jurcău et al., 2022).

Of note, atherosclerosis (AS) and cardiometabolic disorders such as obesity and type 2 diabetes (T2D) are potential risk factors for the development and progression of AD through the induction of systemic inflammation and oxidative stress (Gustavsson et al., 2020; Kacirova et al., 2020). Besides, AD increases the risk of AS, T2D, and obesity through augmentation of Aβ, which crosses the blood–brain barrier (BBB) into the systemic circulation (Lupaescu et al., 2022; Meakin et al., 2020; Nordestgaard et al., 2022; Toledano et al., 2024). Peripheral Aβ, by the generation of endothelial dysfunction and insulin resistance (IR), contributes to the progression of AS, T2D, and obesity (Clarke et al., 2015; Gupta & Iadecola, 2015; Shigemori et al., 2022; Xu et al., 2023). Surprisingly, Aβ, which is also produced peripherally in the pancreas, skeletal muscles, and adipose tissues, contributes to the development of AD (Y. Guo et al., 2021). Thus, Aβ seems to be a potential link between AD and AS. Therefore, this review aims to discuss the causal relationship between AS and AD.

## The Causal Relationship Between AD and AS

### AS Increases AD Risk

**Fig. 3:**
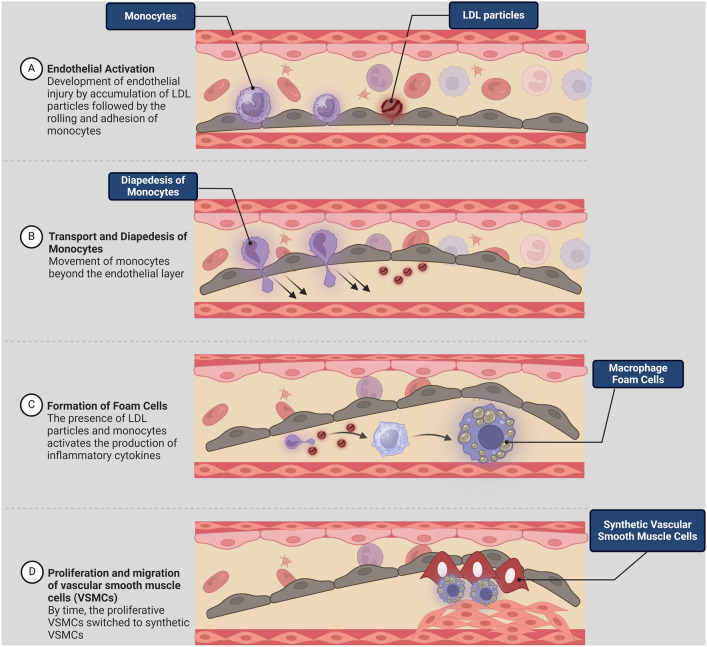
Pathogenesis of AS**:** Endothelial injury by the accumulation of LDL activates the expression of adhesion molecules and monocyte diapediasis. These inflammatory changes trigger macrophage activation and the formation of foam cells. Furthermore, foam cells and the proliferation of vascular smooth muscle cells form atherosclerotic plaque (AP)

AS is a chronic inflammatory disease characterized by the narrowing of the arterial lumen due to the deposition of oxidized cholesterol in the intima of arteries and the formation of atherosclerotic plaques (AP) (Al-Kuraishy et al., 2021, 2024; Alomair et al., 2024; Alsaidan et al., 2023; Alshehri et al., 2024; Turkistani et al., 2024). AP comprises lipid-laden macrophages, hypertrophied smooth muscle cells, and inflammatory cells (Libby, 2021). AS is started at a young age and becomes asymptomatic, though severe narrowing of arteries leads to many clinical manifestations, such as stroke, ischemic heart disease, and kidney dysfunction (He et al., 2024a, 2024b). The potential risk factors for AS are multifactorial, such as dyslipidemia, T2D, obesity, hypertension, smoking, high-fat diet, and sleep disorders (He et al., 2024a, 2024b; Full et al., 2023). The pathogenesis of AS is complex and related to the development of endothelial injury, transport and diapedesis of monocytes, formation of foam cells, and proliferation and migration of vascular smooth muscle cells (Fig. 3).

Of note, vascular-related dementias, which include vascular dementia and unspecified dementia, are often called non-AD dementia and are commonly associated with cardiometabolic disorders such as AS because of shared mechanisms among them (Nordestgaard et al., 2022). In addition, AS and T2D are interrelated in the pathogenesis of AD and other dementia types (Mohseni-Moghaddam et al., 2022). Numerous studies revealed an association between cerebral AS and AD risk. A prospective population-based longitudinal study illustrated that midlife AS is associated with the risk of late-onset dementia. Notably, high carotid intima-media thickness is correlated with high CSF vascular dementia biomarkers (Gustavsson et al., 2020). A case–control study illustrated that the abnormality of the circle of Willis detected by brain MRI is linked with the development of AD (Roher et al., 2011). A cohort study observed that patients with severe intracranial AS are associated with the development of AD (D. Zhao et al., 2024).

Furthermore, patients with carotid AS are linked with the development of cognitive impairment and subcortical ischemic vascular dementia (Liu et al., 2021). Remarkably, about 50% of AD cases globally are possibly attributable to many vascular risk factors, including AS. Reducing vascular risk factors by treating the underlying causes with specific treatment reduces AD risk by 10–25% (Barnes & Yaffe, 2011). However, other clinicopathological studies found no association between cerebral AS and AD (Dolan et al., 2010; Zheng et al., 2013). Longitudinal research has shown that intracranial AS was associated with an elevated risk of dementia but not with the risk of AD (Dolan et al., 2010).

Furthermore, brain autopsies from a longitudinal study revealed that cerebral AS was associated with the risk of multi-infarct dementia but not with AD neuropathology (Zheng et al., 2013). This discrepancy is attributed to the heterogeneity of studies and differences in the selection of patients with dementia (Toledo et al., 2013). Notwithstanding these contradictory results, a systematic review and meta-analysis including 13 clinical research indicated that AS is often linked to the onset and progression of AD (Xie et al., 2020).

In addition, examining several studies on focal ischemia injuries and chronic cerebral hypoperfusion in rat models may be advantageous, as it demonstrated a notable elevation in the translation levels of amyloid precursor protein (Kalaria et al., 1993; Z. Zhao et al., 2021; Pawar & Pardasani, 2024). This elevation generally results from the aggregation of amyloid-beta peptides within the brain’s parenchyma. Furthermore, chronic vascular insufficiency has been noted to induce the cleavage of APP into pieces comparable in size to Aβ in these rodent models (Bennett et al., 2000). It has been shown that cerebral AS is a potential risk for the development of AD and dementia (Gustavsson et al., 2020). Cerebral AS and associated chronic cerebral hypoperfusion and hypoxia promote Aβ production and increase AD neuropathology (Iadecola, 2010). Cerebral AS inhibits the clearance of Aβ via damage to the brain’s glymphatic and perivascular pathways by inducing oxidative stress and neuroinflammation (Wei et al., 2023). In a chronic blood artery occlusion paradigm, researchers observed a gradual accumulation of Aβ peptides in aged rats, underscoring age-related susceptibilities in the brain. The patterns of Aβ deposition showed a steady shift from neuronal cells to the extracellular matrix, closely mirroring the traits linked to sporadic AD. The response to hypoxia is believed to be associated with substantial elevations in the activity of specific amyloidogenic proteases, notably β- and γ-secretases, which are accountable for APP processing (Salminen et al., 2017). Conversely, the activity of the non-amyloidogenic α-secretase seems to diminish in this environment. These results illuminate the intricate biochemical mechanisms associated with vascular-related cognitive loss and the etiology of AD (Lahiri et al., 2003; Polis & Samson, 2019).

Besides, Aβ, through induction of vascular inflammation and endothelial dysfunction, provokes cerebral amyloid angiopathy (CAA) and AS (Iadecola, 2010). CAA is common in patients with brain micro-infarcts due to cerebral AS (Soontornniyomkij et al., 2010).

Moreover, CAA-induced chronic cerebral hypoperfusion and hypoxia stimulate Aβ production and reduce Aβ clearance with subsequent exaggeration of CAA and cognitive impairment (Attems et al., 2011). Interestingly, chronic cerebral hypoperfusion and hypoxia activate the expression and the activity of β and γ secretase enzymes with subsequent exaggeration of amyloidogenic processing of APP and generation of neurotoxic Aβ (L. Li et al., 2009). Numerous experimental investigations have shown that prolonged cerebral hypoperfusion and hypoxia lead to cognitive impairment and AD neuropathology (Faraco et al., 2016; Okamoto et al., 2012).

Moreover, AS, through induction BBB injury, may induce the pathogenesis of AD. Also, AS-induced ischemic stroke triggers BBB injury and the transport of Aβ into the CNS (ElAli et al., 2011; R. Chen et al., 2021).

**Fig. 4:**
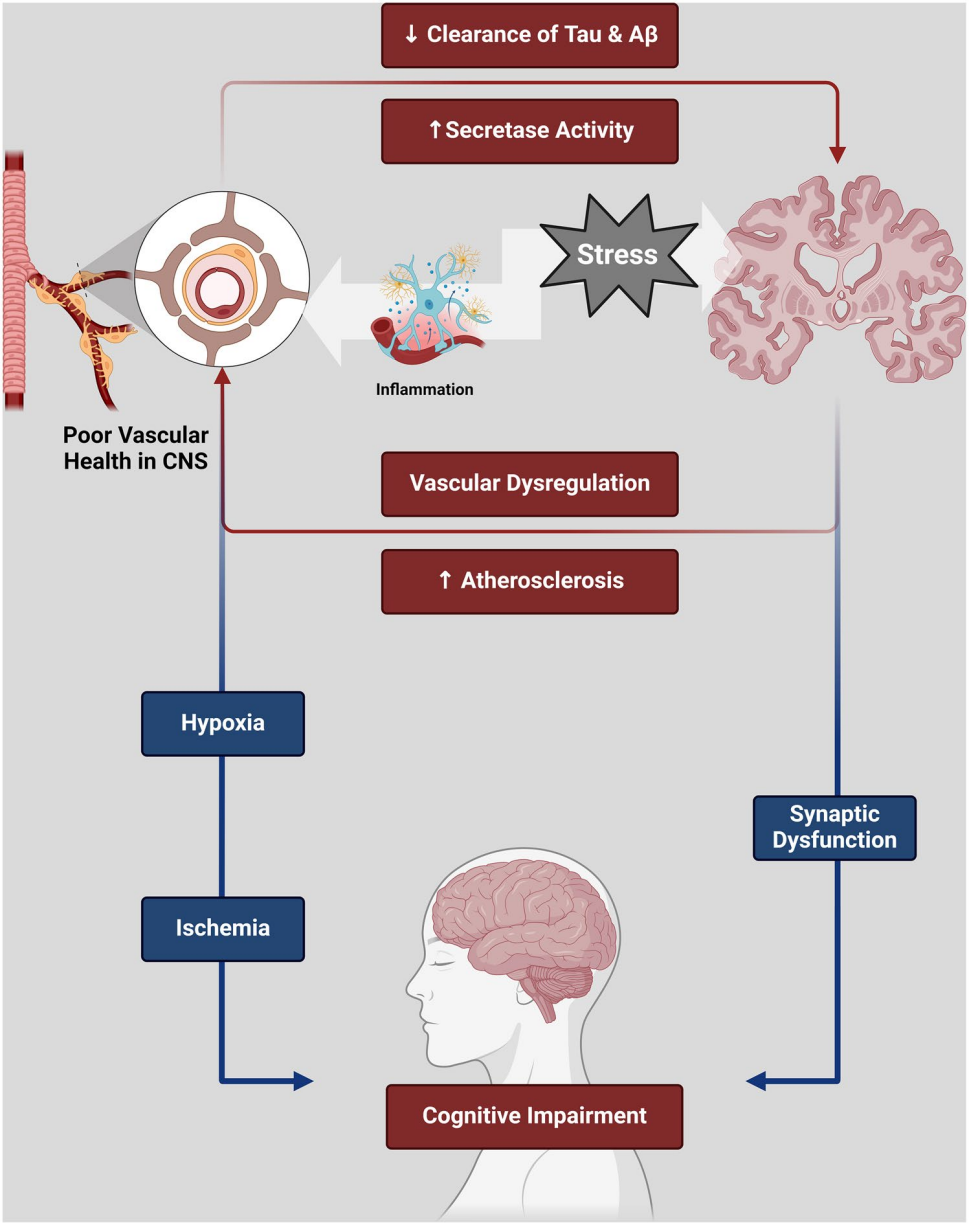
Overlap between AD and AS: Cerebral vascular dysfunction increases the production of Aβ by activating γ and β secretases with impairment of Aβ clearance, resulting in the development of AD. In addition, ischemia and hypoxia in cerebral vascular dysfunction trigger the development of synaptic dysfunction and the progression of cognitive impairment. In turn, AD neuropathology, by augmenting peripheral Aβ, increases AS development

**Fig. 5:**
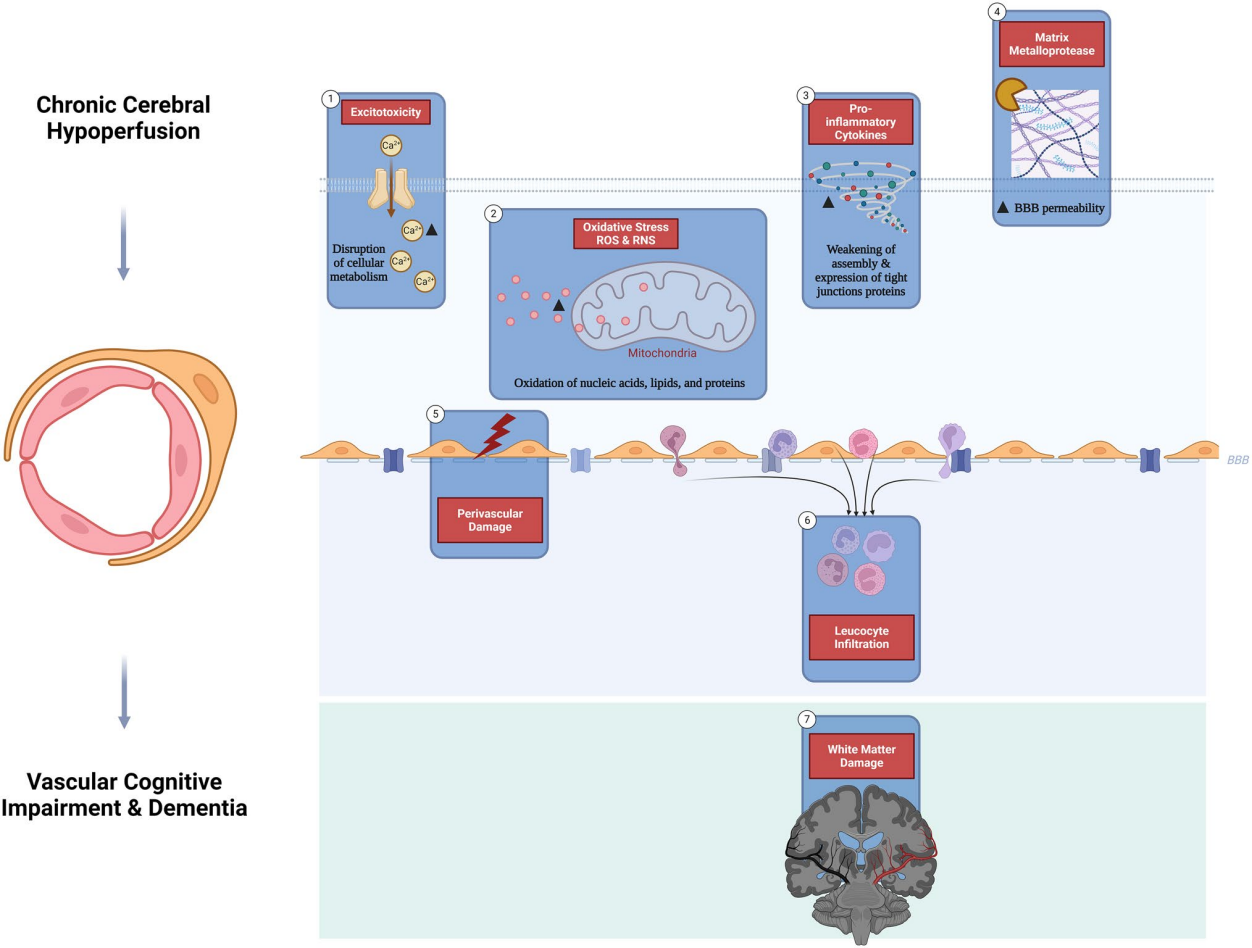
Chronic cerebral hypoperfusion and risk of AD: Chronic cerebral hypoperfusion is involved in the development of vascular cognitive dysfunction and dementia. Chronic cerebral hypoperfusion leads to the development of excitotoxicity, oxidative stress, activation, the release of pro-inflammatory cytokines, and matrix metalloproteinase (MMPs). These pathological changes induce BBB injury and infiltration of leucocytes with subsequent neurodegeneration in AD

These observations suggest an overlap between AD and AS. It has been proposed that AS and associated hypertension and previous myocardial infarction prompt the development of dementia (Fig. 4) (Stern & Frishman, 2024). These verdicts suggested that AS facilitates the establishment and progression of AD by inducing chronic cerebral hypoperfusion and hypoxia (Fig. 5).

### AD increases AS Risk

Historically, it is noteworthy that Dr. Alzheimer’s inaugural autopsy of an Alzheimer's disease patient disclosed considerable brain shrinkage and indications of arteriosclerosis (Pearce, 2000). Mounting data show that persistent reduced blood flow to the brain, which commonly accompanies aging, comes from cerebral atherosclerosis and endothelial dysfunction (de la Torre, 2012). The two interrelated pathogenic processes might result in a crisis in the cerebral energy supply, eventually precipitating the hallmark neurodegeneration seen in Alzheimer's disease. Rising evidence highlighted that AD neuropathology contributes to the pathogenesis of AS by increasing circulating Aβ, which induces endothelial dysfunction (C. Chen et al., 2024). AD and AS are often interrelated due to shared similarities in the development of oxidative stress and inflammation (Tini et al., 2020). It has been stated that Aβ may induce the progression of AP by inducing oxidative stress and inflammation (Kisler et al., 2017). It has been reported that Aβ is implicated in the formation of AP and the pathogenesis of AS by activating intimal macrophage and inducing endothelial injury. Therefore, neuronal Aβ in AD is correlated with the severity of fatty streak of AS in transgenic mice by triggering an inflammatory response in vascular endothelium (Tibolla et al., 2010).

Notably, the BBB protects neurons from factors in systemic circulation and maintains the highly regulated CNS internal milieu, which is required for proper synaptic and neuronal functioning (Wu et al., 2024). BBB disruption allows influx into the brain of neurotoxic blood-derived debris, cells, and microbial pathogens. It is associated with inflammatory and immune responses, which can initiate multiple pathways of neurodegeneration (Shin et al., 2019). BBB dysfunction is a typical AD due to the deposition of Aβ, which initiates a cascade of molecular events that cause neurodegeneration, leading to AD onset and progression. Aβ perturbs the transporters and secretion of neurotoxic substances by the BBB (Shin et al., 2019; Wu et al., 2024). However, BBB injury may be the primary event in the pathogenesis of AD. Findings from preclinical studies demonstrated that microvascular injury plays a key role in the pathogenesis of AD after mild traumatic brain injury (Wu et al., 2024). Therefore, restoring vascular functions might be beneficial for patients with mild traumatic brain injury and potentially reduce the risk of developing AD (Hernandez et al., 2022). Moreover, BBB injury is observed in patients with chronic kidney disease and liver disease. Interestingly, the Aβ circulating level increases in chronic kidney and liver disease (Gronewold et al., 2016; D. G. Kim et al., 2016). Hence, increasing Aβ circulating level can cross the deranged BBB, leading to the initiation of AD neuropathology.

Of note, Aβ is efflux from the brain across the BBB into the systemic circulation via low-density lipoprotein receptor-related protein-1 (LRP1). Conversely, a receptor for advanced glycation end products (RAGE) mediates the transport of Aβ from the systemic circulation into the brain. In AD, the concentration of soluble LRP1 and its affinity for Aβ is diminished, leading to an accumulation of Aβ in the brain. Restoration of plasma sLRP1 decreases brain Aβ load in mouse models. However, increasing the expression and the activity of RAGE is associated with exacerbation of neuroinflammation and AD neuropathology (Deane et al., 2009). In addition, LRP1 plays a critical role in regulating endothelial and vascular homeostasis by eliminating atherogenic lipoproteins, such as LDL (J. Chen et al., 2023). Thus, reducing the expression of LRP1 in AD may exacerbate the pathogenesis of AS. Back up to this notion, it has been stated that the expression of LRP1 is reduced in the macrophages, vascular smooth muscles, and peripheral monocytes in patients with subclinical AS (Albertini et al., 2022). However, the expression of RAGE is augmented in AS and contributes to the progression of the inflammatory response, platelet activation, modification of vascular smooth muscles, and endothelial injury in AS (Singh et al., 2022).

Consequently, disruption of the LRP1/RAGE axis is associated with the etiology of both AD and AS. Significantly, different Aβ mixtures in human AP and platelets contribute to the pathogenesis of AS by inducing inflammatory reactions. The origin of Aβ in the AP may be from activated platelets, vascular wall cells, and circulating Aβ (Kokjohn et al., 2011). It has been established that Aβ promotes inflammation in vascular endothelium and progression of AS (Delialis et al., 2024).

Accordingly, a cross-sectional study revealed that high plasma Aβ was associated with carotid AS in non-hypertensive subjects compared to hypertensive patients (C. Chen et al., 2024). Moreover, in hypertensive patients, plasma level of Aβ_1–40_ is also increased (Jiang et al., 2018). Furthermore, Aβ is accumulated with AP of carotid and aortic AS (Bu et al., 2006). In addition, patients with subclinical AS have higher plasma levels of Aβ_1–40,_ which predicts major cardiovascular complications and mortality (Stamatelopoulos et al., 2018). Likewise, elevated plasma Aβ_1–40_ levels are associated with carotid stiffness in individuals with coronary artery disease (CAD) (Stamatelopoulos et al., 2015).

Additionally, the plasma level of Aβ_1–42_ is augmented in the advanced stage of AD patients compared to the early stage of AD patients. In addition, the plasma level of Aβ_1–42_ is higher in ApoE4 carrier AD patients than in non-carriers (Yang et al., 2020). Therefore, ApoE4 status affects the dynamic alterations of peripheral Aβ_1–42_ levels in AD patients. Correspondingly, ApoE4 carrier AD patients had higher AS risk than non-carriers (Beeri et al., 2006), signifying that ApoE4 carrier AD patients due to higher peripheral Aβ_1–42_ level is correlated with the severity of AS. Conversely, a case–control study revealed that plasma Aβ_1–42_ level was reduced in AD patients during the advanced dementia stage, though a further increase in Aβ_1–42_ level is linked with vascular injury (Janelidze et al., 2016). Therefore, alteration of peripheral Aβ occurs in the late stage of AD after the progressive AD neuropathology and high brain amyloid burden. High peripheral Aβ levels in AD contribute to the disturbance of vascular homeostasis by activating von Willebrand factor and factor VIIa, activation of platelets, dysregulation of the activity of protein kinase C (PKC), and increasing the expression of thrombomodulin (Borroni et al., 2002; Carbone et al., 2021; Janelidze et al., 2016). Furthermore, activated platelets are implicated in the pathogenesis of both AD and AS (Carbone et al., 2021; Momi et al., 2022). Activated platelets are identified as the primary source of peripheral APP and Aβ, with platelet activation increasing peripheral Aβ levels (Carbone et al., 2021). In sporadic AD, the peripheral Aβ level is increased due to overproduction of neuronal Aβ (Rajmohan & Reddy, 2017). Peripheral Aβ leads to the activation of platelets and induction of vascular injury through the activation of inflammatory cascades (Garcia-Mejia et al., 2021). These changes result in endothelial dysfunction and initiation of AS vasculopathy.

APP, which increases AD neuropathology, is also expressed in the liver, adipose tissue, and pancreas. Yun et al. (2020) found that plasma APP level was elevated and correlated with cognitive impairment in AD patients. In addition, high plasma APP level in patients with mild cognitive impairment predicts conversion to an overt AD. Dysregulation of APP processing is associated with the development of metabolic disturbances such as obesity, T2D, and AS (Y. Guo et al., 2021). In addition, APP is also expressed in vascular endothelium and contributes to the development of vascular inflammation and the pathogenesis of AS (Austin et al., 2009). It has been shown that overexpression of mutant APP was associated with the development of AS lesions in APPsw transgenic mice (L. Li et al., 2003).

Furthermore, overexpression of mutant APP triggers the development of AS by increasing the adhesion of monocytes to the vascular endothelium in ApoE4-deficient mice (Austin & Combs, 2010). In line with these findings, genetic deletion of the *APP* gene prevents the development of AS in ApoE4-deficient mice (Van De Parre et al., 2011). These findings indicated that increasing peripheral APP in AD could be a possible risk factor in the development and progression of AS. Notably, peripheral APP processing in AS is mainly through the amyloidogenic pathway due to higher expression of peripheral β-secretase enzyme (Pennington & DeAngelis, 2016; Zuliani et al., 2020). The activity of β-secretase is augmented in serum and the brain in patients with late-onset AD and vascular dementia (Zuliani et al., 2020). Findings from in vitro study demonstrated that the activity of β-secretase is accelerated and participates in the development of vascular lesions (Meakin et al., 2015). In addition, the expression of β-secretase correlates with the severity of AS (Gergiopoulos et al., 2019). Besides, the activity of γ-secretase is also associated with the pathogenesis of AS in mice (K. Kim et al., 2020).

Nonetheless, the expression of α-secretase (ADAM10), which facilitates the processing of APP via a non-amyloidogenic route, is diminished in AS (van der Vorst et al., 2018). Notably, α-secretase regulates endothelial function, leukocyte adhesion, binding of membrane-bound proteins, and angiogenesis. A deficiency of α-secretase induces the development of the AP by increasing ox-LDL uptake and induction of endothelial inflammation in ADAM10-deficient mice (van der Vorst et al., 2023). Therefore, α-secretase has an atheroprotective effect against the development of AS. A case–control study illustrated that polymorphism of the *ADAM10* gene is associated with an increasing risk of AS in the Chinese population (You Li et al., 2013). Therefore, disturbance of peripheral APP processing in AD may induce the development of AS. Thus, AD augments AS risk by increasing Aβ levels and disrupting APP processing. AD is considered a possible risk factor for the onset and advancement of AS. Hence, AD patients should be investigated and screened for the risk of AS.

Of interest is the dysregulation of the immune system, which is a cardinal feature of AD, and a considerable body of evidence indicates pathological alterations in central and peripheral immune responses in AD (Herrera-Rivero et al., 2019). Besides, immune dysregulation has been observed in the brain and blood of patients with AD. However, a convenient assay to evaluate peripheral immune dysregulation in AD has not been developed, partly due to inconsistent observations from different studies. A case–control study showed significant peripheral immune dysregulation in AD patients compared to healthy controls (Z. Guo et al., 2019). AD, regarded as a systemic immune process, raises important questions about how communication between the peripheral and central compartments occurs and whether this crosstalk represents a therapeutic target. A case–control study revealed that peripheral inflammatory biomarkers reflecting immune dysregulation were higher in AD patients than healthy controls (Delaby et al., 2015). It has been shown that neuroinflammation, which is associated with AD neuropathology, has been presumed to be a response to pathophysiological events in AD. A case–control study illustrated that the association between neuroinflammation and AD is more robust in young AD patients than in the oldest patients (Hoozemans et al., 2011). This age-dependent relationship between inflammation and the clinical phenotype of AD has implications for the interpretation of biomarkers and treatment of the disease. However, data from many studies have established that immune system-mediated actions contribute to and drive AD pathogenesis. It has been established that neutrophils infiltrate the AD brain parenchyma when acute colitis occurs, and this infiltration is significantly related to disease progression in transgenic mice (Kaneko et al., 2023).

Similarly, immune dysregulation is implicated in the pathogenesis of AS. Widespread research in preclinical models and emerging evidence in humans have established the crucial roles of the innate and adaptive immune systems in driving AS-associated chronic inflammation in arterial blood vessels. A prospective cohort study found that an increased granulocyte count was associated with a higher risk of atherosclerotic cardiovascular disease in the general population. Moreover, higher levels of granulocytes were associated with larger volumes of arterial calcification. Arterial calcifications may explain a proportion of the link between granulocytes and AS (Fani et al., 2020). Immune cell activation modulates atherogenesis and provides an update on the contributions of innate and adaptive immune cell subsets in AS (Libby et al., 2013). Moreover, peripheral and central immune dysregulation in AD and AS augment the accumulation of Aβ in the brain and AP. Hence, immune dysregulation could be a potential link in the pathogenesis of AD and AS.

Central and peripheral Aβ could be a potential link between AD and AS. Therefore, targeting of central and peripheral Aβ may prevent the development and progression of AD and AS. However, the present review had several limitations, such as the molecular mechanism underlying the effect of Aβ in both AD and AS was not discussed. Hence, further preclinical and clinical studies are recommended in this regard.

## Conclusion

It is well known that AS can induce the development and progression of AD through chronic cerebral hypoperfusion and cerebral hypoxia that induce APP processing and generation of Aβ. In addition, AS impedes the clearance of Aβ from the brain into the systemic circulation, leading to the augmentation of the brain's amyloid load. Additionally, AD is a possible risk factor for the onset and advancement of AS via inducing systemic inflammation and oxidative stress through Aβ. Peripheral Aβ induces endothelial dysfunction, hence facilitating the progression of AS. Consequently, Aβ is a potential connection between AD and AS.

Taken together, there is a closely interrelated relationship between AS and AD. AS promotes the development of AD, which also increases the risk of AS.

Although observational and experimental evidence suggests a connection between AD and AS, this link between AD and AS remains ambiguous, particularly regarding the role of Aβ in vascular injury and the involvement of APP processing in atherogenesis. These hypotheses need human and longitudinal empirical research for validation. Numerous studies have associated AS with AD via mechanisms of inflammation and oxidative stress, whereas other research indicates negligible or absent correlations between cerebral AS and AD neuropathology. This disparity necessitates further examination of molecular mechanisms linking these diseases. Limited information exists about the impact of peripheral Aβ on endothelial dysfunction and AS. Animal models provide valuable insights; human studies are necessary to establish causation. Therefore, additional studies are recommended to elucidate the molecular mechanisms underlying the association AS and AD.

## Data Availability

No datasets were generated or analyzed during the current study.
